# The recent two decades of traumatic brain injury: a bibliometric analysis and systematic review

**DOI:** 10.1097/JS9.0000000000001367

**Published:** 2024-04-11

**Authors:** Ziyin Ye, Zhi Li, Shiyu Zhong, Qichen Xing, Kunhang Li, Weichen Sheng, Xin Shi, Yijun Bao

**Affiliations:** a Department of Neurosurgery, The Fourth Hospital of China Medical University, Huanggu; b Department of Oncology, The First Hospital of China Medical University, Heping; c School of Health Management, China Medical University, Shenyang, People’s Republic of China

**Keywords:** bibliometric analysis, Latent Dirichlet Allocation, topic modeling, traumatic brain injury

## Abstract

**Background:**

Traumatic brain injury (TBI) is a serious public health burden worldwide, with a mortality rate of 20–30%; however, reducing the incidence and mortality rates of TBI remains a major challenge. This study provides a multidimensional analysis to explore the potential breakthroughs in TBI over the past two decades.

**Materials and methods:**

The authors used bibliometric and Latent Dirichlet Allocation (LDA) analyses to analyze publications focusing on TBI published between 2003 and 2022 from the Web of Science Core Collection (WOSCC) database to identify core journals and collaborations among countries/regions, institutions, authors, and research trends.

**Results:**

Over the past 20 years, 41 545 articles on TBI from 3043 journals were included, with 12 916 authors from 20 449 institutions across 145 countries/regions. The annual number of publications has increased 10-fold compared to previous publications. This study revealed that high-income countries, especially the United States, have a significant influence. Collaboration was limited to several countries/regions. The LDA results indicated that the hotspots included four main areas: ‘Clinical finding’, ‘Molecular mechanism’, ‘Epidemiology’, and ‘Prognosis’. Epidemiological research has consistently increased in recent years. Through epidemiological topic analysis, the main etiology of TBI has shifted from traffic accidents to falls in a demographically aging society.

**Conclusion:**

Over the past two decades, TBI research has developed rapidly, and its epidemiology has received increasing attention. Reducing the incidence of TBI from a preventive perspective is emerging as a trend to alleviate the future social burden; therefore, epidemiological research might bring breakthroughs in TBI.

## Introduction

Highlights

- Traumatic brain injury (TBI) is an enormous public health challenge with high incidence and mortality rate.
- We used bibliometric and Latent Dirichlet Allocation analyses to analyze publications focusing on TBI.
- The hotspots of TBI include four main areas: ‘Clinical finding’, ‘Molecular mechanism’, ‘Epidemiology’, and ‘Prognosis’, and its epidemiology has received the most attention; therefore, epidemiological research might bring breakthroughs for TBI.

Traumatic brain injury (TBI) is an enormous public health challenge that results in health loss and disability for individuals, and represents a burden on the healthcare system and the economy through lost productivity and high healthcare costs^1^. Trauma is the leading cause of death in people aged 1–45, with most of the deaths caused by TBI. More than 50 000 deaths occur due to TBI in the United States each year^2,3^. TBI can be clinically classified as mild, moderate, or severe according to the Glasgow Coma Scale (GCS), and the associated permanent disability rates are 10%, 60%, and 100%, respectively, with overall mortality rates of 20–30%^4^. The burden of TBI is evident worldwide and particularly pronounced in low-income countries and middle-income countries (LMICs). These countries face more risk factors that contribute to TBI and have ill-prepared health systems to cope with the associated health consequences^5^. The implementation of various measures has contributed to the prevention and treatment of TBI^5^. Therefore, the global analysis and oversight of TBI publications allows scholars to quickly focus on hotspots in the field of TBI. Our current research attempts to explore progression of prevention, diagnosis, and treatment, which are more effective to reduce the social burden of TBI.

Based on available data, our study provides a comprehensive analysis of research trends in the field of TBI from 2003 to 2022. Qualitative and quantitative analyses of the literature revealed research hotspots and frontiers in this field. VOSviewer and bibliometric online platforms are currently widely used, allowing the analysis of raw data downloaded from databases^6^. In particular, we used Latent Dirichlet Allocation (LDA) in our study as a deep learning method for topic modeling^7,8^, which is an algorithm for analyzing the hidden structure presented in a collection of documents and automatically identifying topics^9^.

The methods above permits a more scientific interpretation of the research in the TBI field, including several studies in neuromonitoring^10^, rehabilitation^11^, pediatric TBI^12^, mild TBI^13^, and severe TBI^14^. Recently, *The Lancet Neurology* had an online article that reviewed the articles published in this journal^15^, but there is a lack of comprehensive bibliometric analysis of the TBI field to clearly state the gap in the study that pave the way for it to be carried out. Topic modeling was performed using LDA to identify research hotspots. This study analyzed publications in the TBI field over the last two decades using data from the Web of Science Core Collection (WOSCC), aiming to reveal research trends, hotspots, and frontiers.

## Material and methods

### Search strategy

**Figure 1:**
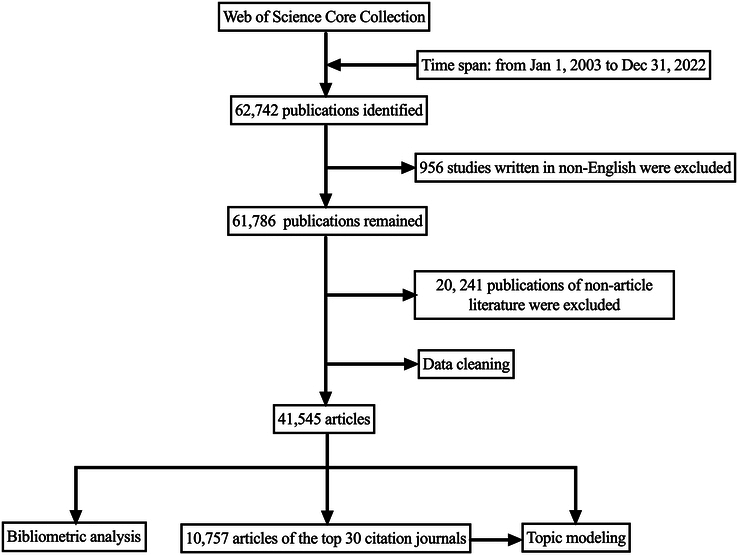
Literature search and selection.

The data used in the analysis were obtained from the WOSCC, which can provide the most comprehensive data for the bibliometric analysis. To obtain complete literature data on TBI, a comprehensive search of the WOSCC was conducted with the following strategy: TS=(‘Brain Injury, Traumatic’ OR ‘Traumatic Brain Injuries’ OR ‘Trauma, Brain’ OR ‘Brain Trauma’ OR ‘Brain Traumas’ OR ‘Traumas, Brain’ OR ‘Encephalopathy, Traumatic’ OR ‘Encephalopathies, Traumatic’ OR ‘Traumatic Encephalopathies’ OR ‘Injuries, Brain, Traumatic’ OR ‘Traumatic Encephalopathy’ OR ‘Traumatic Brain Injuries’ OR ‘Brain Injuries, Traumatic’ OR ‘Craniocerebral Trauma’). The time span was from 2003 to 2022, the language in the search formula was limited to English, and the type of literature was limited to ‘Article’. The search was completed independently by two researchers on 6 January 2023, and data analysis was performed after consistent results were confirmed. A flowchart of this process was shown in Figure 1.

### Data collection and methods

#### Bibliometric analysis

We performed a bibliometric analysis using VOSviewer (1.6.18, Leiden University), an online bibliometric platform (Bibliometric.com), CiteSpace (5.8. R3, Drexel University), SCImago Graphica (Beta 1.0.28), and R software (version 4.2.1). The analyses included the number of publications, co-authors, and cocitations by country, institution, author, and keyword visualization. Co-authorship analyses and clustering with keywords were conducted using VOSviewer and SCImago Graphica. After the input of the downloaded data into VOSviewer, we arranged and selected options such as ‘co-authorship’, ‘co-occurrence’ and ‘author’ in the panel to fulfill our research objectives, and then limited the minimum number of documents so that the figures can present 50–60 thresholds. We output the results and used SCImago Graphica for the secondary mapping for information simplification and visualization. Keywords bursts were analyzed and visualized using Citespace. Analyses of the core authors, countries, institutions, and journals were constructed on the bibliometric online platform. VOSviewer and SCImago Graphica provide a visualization chart in which different nodes labeled with the detailed information represent authors, institutions, keywords, countries, and journals. The sizes of nodes indicate their corresponding numbers, frequencies, and citations. The links between nodes indicate collaboration, co-occurrence, or cocitation relationships, and the width of link lines indicates the strength of association. Based on the cocitation of journals, the cooperation of authors, institution or countries and the co-occurrence of keywords, VOSviewer, and SCImago Graphica classify the data into different clusters and automatically assigns colors.

#### Topic modeling

We used LDA for topic modeling, which is based on a three-layer Bayesian network that provides document topics in the form of probability distributions. The article titles, abstracts, and keywords were extracted and input into the model corpus. The first 20 prominent feature terms were extracted and generated into one topic, and 30 topics were determined. The naming of each topic was performed by three professors with more than 20 years of clinical and scientific research experience according to the term frequency and literature. Then, we categorized the 30 topics into four broader themes including ‘Clinical finding’, ‘Molecular mechanism’, ‘Epidemiology’, and ‘Prognosis’.

Several metrics are used to determine the number of topics. The Kullback–Leibler divergence method (Arun_2010), proposed by Arun *et al*.^16^, calculates the symmetry between the distribution of variance in the topic-word distribution and the marginal topic distribution. In the pairwise cosine distance method (Cao_Juan_2009), proposed by Cao *et al*.^17^, when the average cosine distance of topics reaches a minimum, the best number of topics based on topic density can be determined. The divergence value is minimal for the optimal number of topics. In the case of model coherence (Coherence_mimno_2011) proposed by Mimno *et al*.^18^, for a number of topics, pointwise mutual information is maximized when the most likely words in a given topic frequently co-occur^19^.

All the above steps were performed independently by two researchers. If the results were inconsistent in the search strategy (e.g. punctuation, date range, and database selection), format of the exported data, and parameters of the visualization tool, a third researcher intervened and discussed to obtain the confirmed data.

#### Statistical analysis

The Mann–Kendall test is a nonparametric test used to identify trends in time-series data. To quantify the temporal trend in the popularity of each topic, the Mann–Kendall statistical method was used according to the proportion of publications in the total annual number of publications.

## Results

### General characteristics of publications

**Figure 2:**
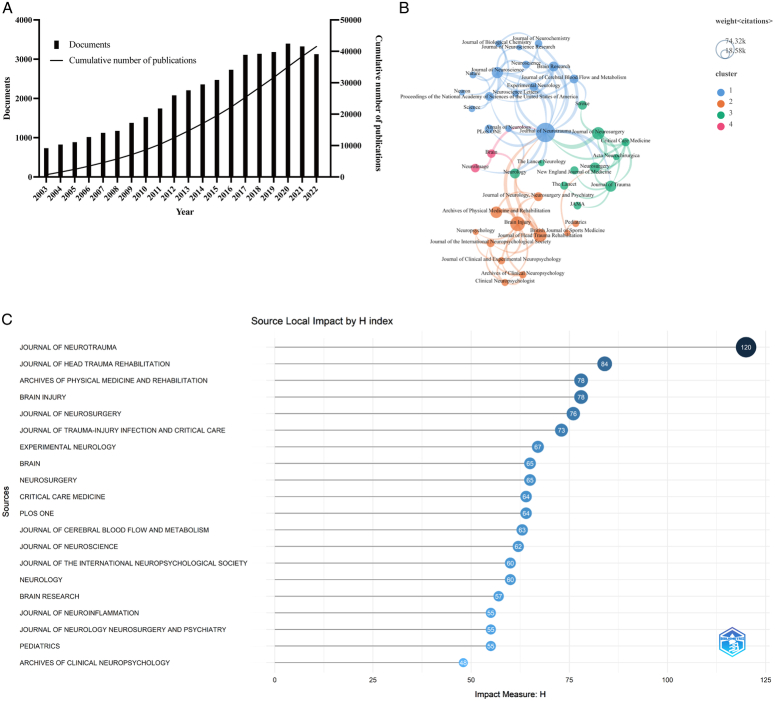
Analyses of publications and journals. (A) Annual publications and the cumulative growth curve from 2003 to 2022. (B) and (C): Analysis of core journals. The top 20 journals with the highest H-index (B) and cocitation network visualization between journals (C). Node represents journal. The different colors symbolize the categories with the variation of cocitation among journals.

The search and selection strategy shown in Figure 1 was implemented and 41 545 original research articles were included. From 2003 to 2022, 12 916 authors from 20 449 institutions in 145 countries/regions worldwide focused on TBI research published in 3043 journals. The statistics of annual publications and cumulative number of publications were shown in Figure 2. The number of publications in this field shows a two-stage trend: a rapid growth stage from 2003 to 2017 and a stable stage from 2018 to 2022. Publications grew rapidly in the first 15 years, from less than 300 to more than 3000 articles per year, and in the past 5 years, the annual number of articles remained stable. These results indicate that research in the field of TBI has reached its highest level of enthusiasm in the last 5 years.

### Productive countries/regions, institutions, and authors analysis

The ranks of the 10 top countries/regions with the largest number of publications were listed in Table 1. The United States was at the top with 21 480 articles, contributing to more than half of the global publications, followed by China with 4481 articles, and Canada with 3313 articles. Based on average citations, the top three countries were the Netherlands with an average of 41.94 citations, the United Kingdom with 41.12, and Switzerland with 35.21. Although China has a large number of publications, the average number of citations remains low, matching the number of publications and implying that the importance of single research should be improved.

**Table 1:** Top 10 countries with the most publications.

Rank	Country	Articles	Citations	Average citations
1	USA	21 480	690 717	32.16
2	China	4681	84 642	18.08
3	Canada	3313	96 822	29.22
4	Australia	2710	80 336	29.64
5	UK	2643	108 678	41.12
6	Germany	1759	58 723	33.38
7	Italy	1290	42 221	32.73
8	Sweden	1021	35 951	35.21
9	Netherlands	973	40 810	41.94
10	France	900	29 337	32.60

The top 10 institutions with the largest number of publications were listed in Table 2. The University of Pittsburgh was the most prolific institution, with the highest number of publications (publication numbers=1114). The University of Washington and the University of Toronto ranked second (publication numbers=955) and third (publication numbers=837), respectively. Institutions with high average citations were ranked at the top with the University of California (average citations=41.63), followed by the University of Pittsburgh (average citations=39.30), and Virginia Commonwealth University (average citations=39.26). Eight of the top ten institutions were from the United States, the most prolific country, and the other two institutions were from Canada and Australia, the third and fourth most prolific countries, respectively. Although China had the second largest number of publications, no institution was ranked in the top 10. These results indicate that high-income countries (HICs) and institutions predominate the field of TBI.

**Table 2:** Top 10 productive institutions.

Institution	County	Articles	Citations	Average citations
University of Pittsburgh	USA	1114	43 780	39.30
University of Washington	USA	955	33 142	34.70
University of Toronto	Canada	837	24 151	28.85
University of Pennsylvania	USA	833	31 401	37.70
Harvard Medical School	USA	729	13 623	18.69
Uniformed Services University of The Health Sciences	USA	726	21 586	29.73
Monash University	Australia	691	22 950	33.21
Virginia Commonwealth University	USA	688	27 013	39.26
Baylor College of Medicine	USA	642	22 739	35.42
University of California, San Francisco	USA	613	25 519	41.63

Authors with high publication volumes were also analyzed, and the top 10 prolific authors were listed in Table 3. The most prolific author was Patrick M. Knochanek from the University of Pittsburgh, with 188 publications and 6000 citations. David K. Menon from the University of Cambridge ranked second with 179 publications and an average citation of 62.94. Seven of the top ten authors were from the United States, implying the dominance and significant contribution of the United States in the field of TBI.

**Table 3:** Top 10 prolific authors.

Rank	Author	Articles	Citations	Average citations	Institution
1	Patrick M. Kochanek	188	6000	31.91	University of Pittsburgh (USA)
2	David K. Menon	179	11 266	62.94	University of Cambridge (UK)
3	Grant L. Iverson	173	5200	30.06	Schoen Adams Res Inst Spaulding Rehabil (USA)
4	Keith Owen Yeates	159	4354	27.38	University of Calgary (Canada)
5	Shari L. Wade	155	4390	28.32	Cincinnati Children’s Hospital Medical Center (USA)
6	Monica S. Vavilala	147	4612	31.37	UW Medicine (USA)
7	H. Gerry Taylor	142	4436	31.24	Case Western Reserve University (USA)
8	Ramon Diaz-Arrastia	137	5404	39.45	University of Pennsylvania (USA)
9	Marek Czosnyka	136	4728	34.76	University of Cambridge (UK)
10	Jennie Ponsford	136	5787	42.55	Monash University (Australia)

### Multiple parameter analysis of core journals

The journal cocitations were analyzed. The core journals (Fig. 2B) were ranked according to the H-index^20^, based on their publication and citation data. The top 10 H-index journals were listed with the number of publications, Journal Citation Report (JCR), impact factors (IF), and JCR partition in 2021 in Table 4. The JCR partition was included to evaluate the influence of journals, because it is more representative than the IF value in a certain field. All the journals in the top 10 were JCR Q1 or Q2 journals.

**Table 4:** Top 10 core journals.

Rank	Journal	Counts	Citations	IF (2021)	H-index	JCR partition
1	*Journal of Neurotrauma*	2553	93 197	4.869	120	Q1
2	*Journal of Head Trauma Rehabilitation*	1119	34 915	3.117	84	Q1
3	*Archives of Physical Medicine and Rehabilitation*	541	21 928	4.06	78	Q1
4	*Brain Injury*	2004	41 120	2.167	78	Q2
5	*Journal of Neurosurgery*	441	20 306	5.526	76	Q1
6	*Journal of Trauma- Injury Infection and Critical Care*	340	17 988	2.961	73	Q1
7	*Experimental Neurology*	319	13 955	5.62	67	Q2
8	*Brain*	130	13 694	15.255	65	Q1
9	*Neurosurgery*	243	15 593	5.315	65	Q1
10	*Critical Care Medicine*	234	12 480	9.296	64	Q1

IF, impact factors; JCR, Journal Citation Report.

The cocitation network of journals in Figure 2C indicated clusters of common research content in the journals. It was divided into four clusters: blue represented neuroscience journals led by the *Journal of Neurotrauma*; orange represented clinical neurology journals led by *Brain Injury*; and green showed mainly high-impact medical journals. The *Journal of Neurotrauma* linked all four clusters of journals, indicating that it has advanced significantly in the field of TBI.

### Cocitation analysis of countries/regions, institutions, and authors

Collaboration among countries/regions, institutions, and authors plays a crucial role in fostering insightful studies and minimizing bias in both basic and clinical research. Therefore, we analyzed collaborations between different countries, institutions, and authors in this study, as shown in SDC Figure 1 (Supplemental Digital Content 1, http://links.lww.com/JS9/C271).

The co-authored publications from the 36 countries were illustrated in SDC Figure 1B (Supplemental Digital Content 1, http://links.lww.com/JS9/C271). Notably, the 10 countries with the highest number of publications exhibited a closer collaborative relationship. The strongest collaboration was with the United States, followed by Australia, Canada, and Europe. In addition, 35 institutions with more than 350 publications were included in the analysis. According to the co-authorship analysis in SDC Figure 1C (Supplemental Digital Content 1, http://links.lww.com/JS9/C271), the institutions were categorized into four clusters with different colors. The blue, pink, and green clusters predominantly represented institutions from the United States, and the pink cluster primarily comprised Harvard University and its affiliated hospitals, and Boston University, which are located in Boston. The orange cluster comprises institutions in Europe, Canada, and Australia. These findings underscore the dominant position of the United States in TBI research, along with the strong collaboration between Australia, North America, and Europe. This finding was consistent with observations in the collaboration analysis of countries, implying that collaboration in TBI research is limited. Therefore, international cooperation among global institutions should be enhanced.

The author co-authorship map (SDC Figure 1A, Supplemental Digital Content 1, http://links.lww.com/JS9/C271) revealed that influential authors such as Patrick M. Kochanek, David K. Menon, Keith Owen Yeates, Shari L. Wade, and H. Gerry Taylor, were dominant. Authors in a single cluster collaborate closely; however, few connections exist between different clusters. To enhance the prevention, treatment, and outcomes of TBI, various international institutions have collected diverse data on patients with TBI, including initiatives such as InTBIR (https://intbir.incf.org/), CENTER-TBI (https://www.center-tbi.eu/), and TRACK-TBI (https://tracktbi.ucsf.edu/)^3^. Notably, the core members of these databases are leaders in the fields mentioned above (SDC Figure 1A, Supplemental Digital Content 1, http://links.lww.com/JS9/C271). For instance, Andrew I. R. Maas from the University Hospital Antwerp and David K. Menon from Cambridge University served as key persons in CENTER-TBI. Kevin K. W. Wang and Ramon Diaz-Drastia were the biological and clinical leaders of TRACK-TBI, whereas David O. Okonkwo, David K. Menon, Hester F. Lingsma, and Geoffrey T, Manley. Manley were the core members of InTBIR. In recent years, China, represented by Dr Jiyao Jiang, has also joined the CENTER-TBI^5^, contributing a large amount of patient data for international collaboration and providing a unique opportunity for comparative analysis between Chinese and European populations.

### Keyword analysis

**Figure 3:**
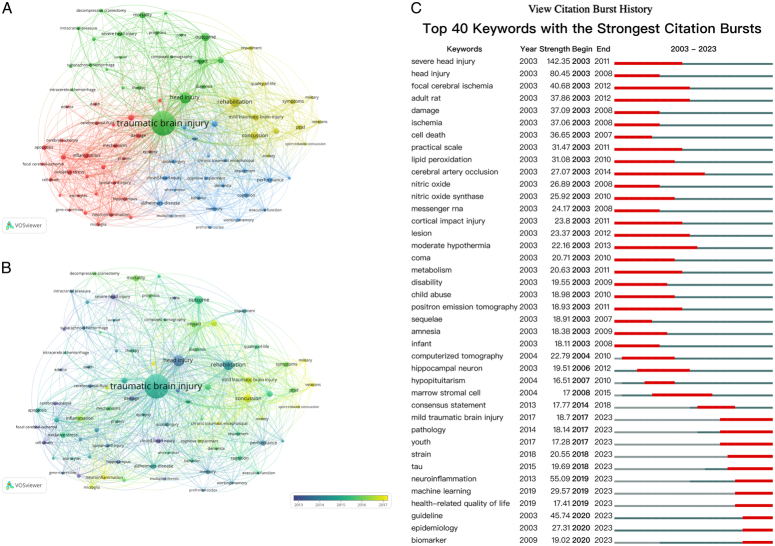
Distribution of keywords. Node represents keyword. (A) Clustering map of keywords. The different colors indicate the separate categories based on the co-occurrence of keywords. (B) Clustering map of keywords according to the average time of appearance. The gradient colors represent the average time of appearance of different keywords, and the darker colors indicate the earlier period, while the lighter colors mean the more recent period. (C) Keywords bursts clustering map according to the average time of appearance.

The keyword analysis illustrates the core fields and hotspots of research. After removing the duplicates, we extracted 66 641 keywords from 41 545 articles, among which 84 keywords that appeared more than 600 times were constructed in two visualization maps (Fig. 3): a network that grouped keywords into different clusters by different colors (Fig. 3A), and an overlay visualization map based on their occurrence patterns over time (Fig. 3B). Figure 3C showed the top 40 keywords with the strongest citation bursts; the red bars indicate the bursts period, including the start and end years.

For keyword co-occurrence (Fig. 3A), the keywords were divided into four clusters: the red cluster was primarily centered on the pathophysiological mechanisms of TBI. The key terms included “neurodegeneration”, “neuroinflammation”, “neuroprotection”, and “neurosurgery”. Notably, “neuroinflammation” and “microglia” emerged after 2017 with a strong correlation, indicating their significance as recent popular research topics. In addition, “biomarkers” gained prominence after 2017, suggesting that biomarker research is a recent hotspot. The green cluster was predominantly related to the clinical treatment of TBI and encompassed keywords such as ‘decompressive craniectomy’, ‘Glasgow coma scale’, and ‘intracerebral hemorrhage’. The strong correlation between ‘outcome’ and ‘mortality’ suggested that the high mortality rate associated with TBI remains a significant challenge in this research field. This finding was consistent with the current epidemiology of TBI. Keywords in the blue cluster were mainly associated with neurological complications, including ‘alzheimers-disease’, ‘axonal injury’, and ‘cognitive impairment’, which were mostly the clinical manifestations of chronic TBI. The yellow cluster centered on TBI prognosis included keywords related to mental illness, such as ‘PTSD’, ‘anxiety’, and ‘depression’. Notably, ‘mild traumatic brain injury’ (mTBI) was strongly correlated with ‘mental trauma’, suggesting a higher likelihood of psychiatric disorders following mTBI. Terms such as ‘military’, ‘veterans’, and ‘sport-related concussion’ emerged after 2017 indicating an increasing research focus on these areas in the TBI field. The gradual shift in research focus from ‘severe head-injury’ to ‘mild traumatic brain injury’ was shown in Figure 3B, which potentially reflected changes in the epidemiology of TBI. Moreover, the keywords bursts depicted in Figure 3C were consistent with the aforementioned trends, demonstrating a decline in research on severe TBI (sTBI) and a shift in focus toward mTBI. Epidemiological research on TBI warrants further attention.

### Topic modeling by LDA

**Figure 4:**
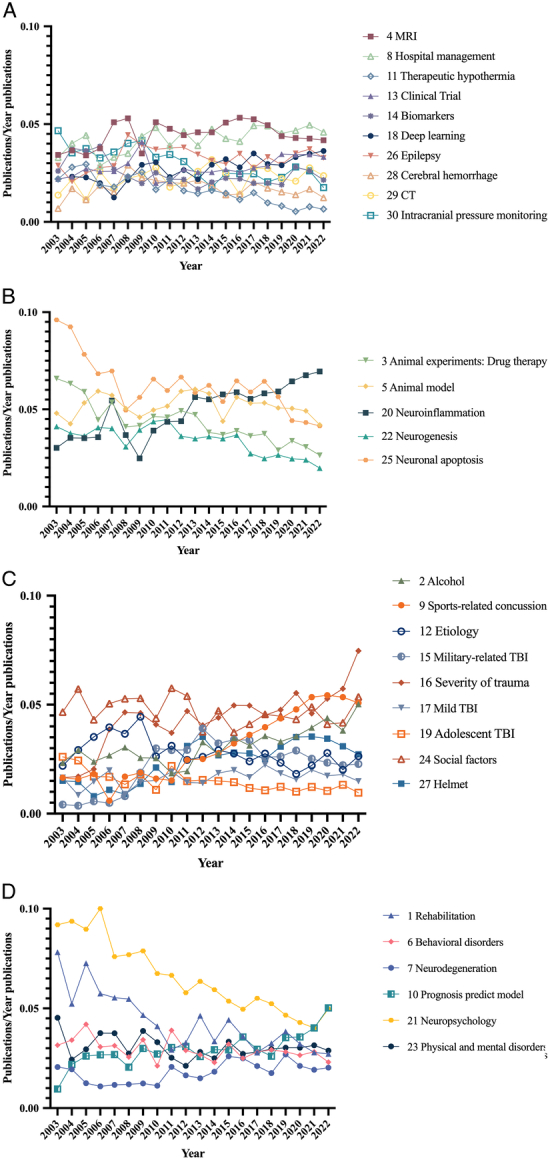
Topic trends of the overall data from 2003 to 2022. (A) Clinical finding; (B) Molecular mechanism; (C) Epidemiology; (D) Prognosis.

**Figure 5:**
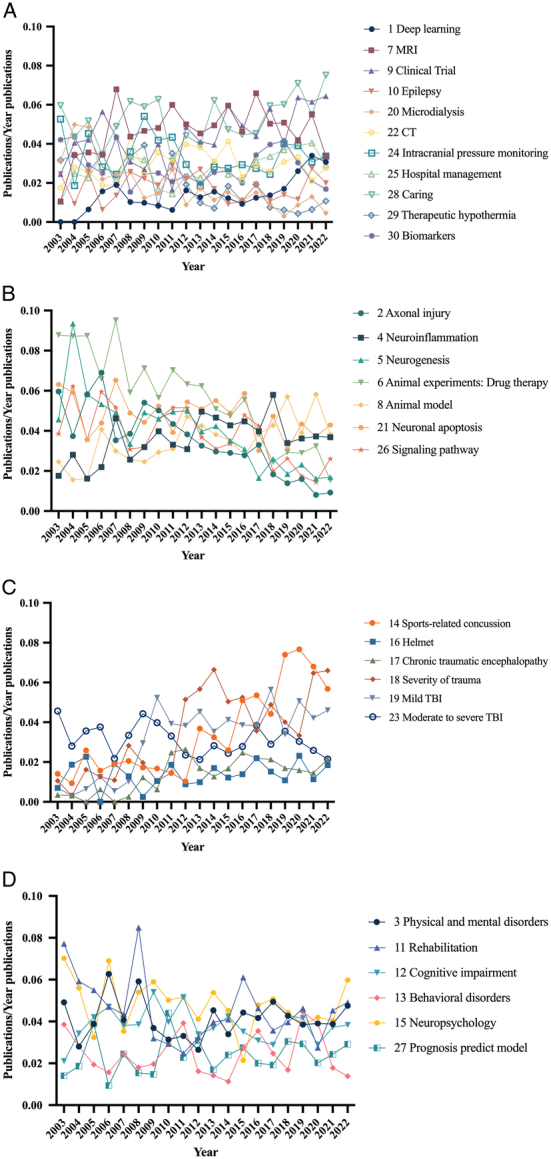
Topic trends of the top 30 citation journals data from 2003 to 2022. (A) Clinical finding; (B) Molecular mechanism; (C) Epidemiology; (D) Prognosis.

To provide a more accurate analysis of the research hotspots, a topic modeling analysis of the databases and data from the top 30 citation journals including 10 757 articles was conducted. It was possible to identify the topics studied by scholars in all included TBI literature and the topics of interest in highly cited articles and to compare their potential differences. After optimizing the data, 20 prominent feature words for each topic were selected with the removal of meaningless terms such as ‘Outcome’, ‘Improve’, and ‘Compare’. The changes in the model comparison metrics were shown in SDC Figure 2 (Supplemental Digital Content 1, http://links.lww.com/JS9/C271). For Arun_2010^16^ and Cao_Juan_2009^17^, the minimum points of the curves were considered. By contrast, the maximum point of the curve was considered for Cohorence_mimno_2011^18^. The number of topics ranged from 25 to 35. Therefore, we identified 30 topics for both the total data (SDC Figure 2A, Supplemental Digital Content 1, http://links.lww.com/JS9/C271) and the data from the top 30 citation journals (SDC Figure 2B, Supplemental Digital Content 1, http://links.lww.com/JS9/C271). The topics were categorized into four groups and named ‘Clinical finding’, ‘Molecular mechanism’, ‘Epidemiology’, and ‘Prognosis’. Annual topic publications were normalized to the total number of annual publications (Figs 4, 5). The Mann–Kendall test was performed to examine the statistics of the trends (SDC Table 1, Supplemental Digital Content 1, http://links.lww.com/JS9/C271).

The results from the overall data (Fig. 4, SDC Table 1, Supplemental Digital Content 1, http://links.lww.com/JS9/C271) indicated that the research focusing on the ‘Clinical finding’ category and the ‘Prognosis’ category remained relatively stable over the two decades. Research on some topics of the ‘Epidemiology’ category increased, whereas others remained unchanged. Most topics exhibited a decreasing trend in the ‘Molecular mechanism’ category. Research on several topics, such as ‘Alcohol’ (Z=3.9906, *P*<0.001), ‘Neurodegeneration’ (Z=2.3035, *P*=0.02), ‘Hospital management’ (Z=3.0822, *P*=0.002), ‘Sports-related concussion’ (Z=4.8991, *P*<0.001), ‘Prognosis predict model’ (Z=3.8609, *P* <.001), ‘Clinical Trial’ (Z=2.4333, *P*=0.02), ‘Severity of trauma’ (Z=4.1853, *P*<0.001), ‘Deep learning’ (Z=3.6013, *P*<0.001), ‘Neuroinflammation’ (Z=5.0938, *P*<0.001), and ‘Helmet’ (Z=3.2120, *P*=0.001) has increased in popularity in recent years. However, topics such as ‘Rehabilitation’ (Z=−4.3151, *P*<0.001), ‘Animal experiments: Drug therapy’ (Z=−4.5746, *P*<0.001), ‘Behavioral disorders’ (Z=−2.1089, *P*=0.04), ‘Therapeutic hypothermia’ (Z=−4.7693, *P*<0.001), ‘Etiology’ (Z=−2.3684, *P*=0.02), ‘Biomarkers’ (Z=−2.1738, *P*=0.03), ‘Adolescent TBI’ (Z=−3.958, *P*<0.001), ‘Neuropsychology’ (Z=−5.0938, *P*<0.001), ‘Neurogenesis’ (Z=−3.9258, *P*<0.001), ‘Neuronal apoptosis’ (Z=−3.7311, *P*<0.001), and ‘Intracranial pressure monitoring’ (Z=−3.6013, *P*<0.001) showed a decreasing trend in popularity. Some topics did not exhibit significant changes (*P*≥0.05), such as ‘MRI’, ‘Mild TBI’, and ‘Social factors’.

The trends observed in the data from the top 30 most-cited journals (Fig. 5, SDC Table 2, Supplemental Digital Content 1, http://links.lww.com/JS9/C271) were similar to those observed in the overall data. The topics of the ‘Molecular mechanism’ category showed a decreasing trend, while those of the ‘Prognosis’ category remained stable in recent years. However, the topics of the ‘Epidemiology’ and ‘Clinical finding’ categories exhibited an increasing trend over the last 5 years. Notably, ‘Deep learning’ (Z=3.1163, *P*=0.002) had rapidly increased in popularity since 2019. Topics such as ‘Neuroinflammation’ (Z=2.3684, *P*=0.02), ‘Animal model’ (Z=3.2769, *P*=0.001), ‘Clinical Trial’ (Z=2.7578, *P*=0.01), ‘Sports-related concussion’ (Z=3.8609, *P*<0.001), ‘Chronic traumatic encephalopathy’ (Z=2.5969, *P*=0.01), ‘Severity of trauma’ (Z=3.212, *P*=.001), ‘Mild TBI’ (Z=3.3418, *P*<0.001), and ‘Hospital management’ (Z=2.2387, *P*=0.03) showed an upward trend over the two decades. Conversely, topics such as ‘Axonal injury’ (Z=−4.6395, *P*<0.001), ‘Neurogenesis’ (Z=−4.1853, *P*<0.001), ‘Animal experiments: Drug therapy’ (Z=−4.7693, *P*<0.001), ‘Microdialysis’ (Z=−3.8609, *P*<0.001), and ‘Signaling pathway’ (Z=−3.0822, *P*=0.002) declined in popularity. Similar to the overall data, some topics did not exhibit significant changes (*P*≥0.05), such as ‘Physical and mental disorders’, ‘Epilepsy’, and ‘Rehabilitation’. Interestingly, ‘Biomarkers’ (Z=−0.876, *P*=0.38) did not show a significant change over the past two decades and experienced a significant decrease in popularity over the past 5 years.

The analysis of the top 30 most cited journals has provided valuable insights into the topical interests of scholars in the field of TBI over the past two decades. The stability of the ‘Clinical finding’ category suggested that clinical research had consistently attracted researchers. These results also indicate that the top 30 most cited journal publications might better reflect the interests of researchers than the overall data. Since the ‘Clinical finding’ and ‘Epidemiology’ categories showed an increasing trend, we discuss them in detail.

## Discussion

TBI is the leading cause of injury-related mortality and disability worldwide, placing a significant burden on patients, their families, and the society^21^. The management of TBI requires specialized rehabilitation, long-term monitoring, and care^22^. In this study, we shed light on advances in TBI research by analyzing publication quantities, journals, countries/regions, co-authorships, and the diverse ranges of topics covered in the literature.

After topic modeling analysis, trends and shifts in various research topics were observed. These trends have highlighted specific topics over the past two decades and revealed the occurrence of novel topics. In addition, some topics exhibited limited change during this period, suggesting the absence of significant breakthroughs in these areas.

This study aimed to provide an overview of the selected topics and identify existing breakthroughs and future topics in TBI research. Our findings show that research related to in-hospital diagnosis and molecular mechanism has stagnated during the TBI disease process. As far as molecular mechanisms of TBI are concerned, several reasons might explain the stagnation of this research area. Firstly, the key mediators are almost already identified in TBI-driven cellular events including a series of responses such as excitotoxicity, mitochondrial damage, oxidative stress, neuroinflammation, and cell death^23^. Secondly, the biomarkers are difficult to detect directly in blood due to the existence of blood-brain barrier, and the cerebrospinal fluid sampling is invasive^24,25^. Thirdly, even the target-engaging, symptom-alleviating, and function-improving findings have been successfully confirmed in animal models of TBI, it is still difficult for the transformation of the discovered mechanisms into clinical trials^26^. In contrast, research on the treatment and epidemiology of TBI, including its etiology, risk factors, and prevention, is emerging as a breakthrough in reducing its social burden caused by TBI.

### TBI is a public health event

#### Epidemiology of TBI: a silent epidemic

TBI is a medical condition and a significant social event, our LDA data shows that ‘Social factors’ as one of the topics of epidemiology is sustained as a hotspot. Unfortunately, many cases of mTBI are underestimated by healthcare professionals or not reported by patients, leading to poor documentation of epidemiological data. Therefore, TBI is considered a ‘silent epidemic’^22^. However, there has been a growing recognition among researchers that reducing the burden of TBI requires epidemiological insights. Our study shows that epidemiology has witnessed an increase in the number of publications over the past two decades.

The causes, incidence, and mortality rates of TBI vary greatly across countries, and even within different regions of the same country. A door-to-door survey conducted in China during the early 1980s reported incidence and mortality rate of 55.4–64.1 and 6.3–9.7 per 100 000 individuals, respectively^27^. These rates were considerably lower than those reported in HICs during the same period, such as the United States (incidence rate of 823.7 per 100 000 individuals) and New Zealand (incidence rate of 811.0 per 100 000 individuals)^2^. The primary causes of TBI reported in China during this period were traffic accidents (31.7%), occupational accidents (23.8%), and injuries from falling objects (21.8%)^28^. Notably, a study on TBI mortality rates revealed a global decrease of ~50% from the 1980s to the early 21st century, with the most significant decline occurring between 1970 and 1990. This decline may be attributed to the introduction of CT scans and intracranial pressure (ICP) monitoring^29^.

With economic development and lifestyle changes, the leading cause of TBI-related deaths in China has shifted. Although limited data are available owing to the absence of a unified TBI registry system, it has been observed that traffic accidents and falls have become more prevalent causes of TBI mortality in China^30,31^. The proportion of TBI cases resulting from traffic accidents increased dramatically from 12.99% in 2002 to 19.68% in 2008^31^. Notably, the mortality rate of TBI increased from 13.23/100 000 in 2006 to 17.06/100 000 in 2008^32^. In contrast, in the United States, where violence-related injuries are common, fatal TBI is predominantly caused by gunshot wounds^33^. The strict prohibition of firearms in China under Chinese law has resulted in significantly fewer cases of violent TBI than in the United States^34^.

Despite numerous studies on epidemiology of TBI, cross-sectional and longitudinal comparisons of morbidity and mortality rates remain challenging. In Europe, the overall incidence rate of TBI ranges from 47.3/100 000 to 898/100 000, with a mortality rate of 3.3 to 28.1/100 000. The leading cause of TBI has shifted from traffic accidents to falls. The incidence of TBI may still increase owing to the widespread motorcycles in LMICs and the rising number of falls among older adults in HICs^21^.

#### Efforts and strategies to reduce TBI risk: a global perspective

Our LDA data indicates the topic hotness of ‘Helmet’ in the past 5 years is higher than before, which shows that countries around the world have implemented various measures to reduce the risk of TBI. In China, significant efforts have been made through policy changes and law enforcement, including enforcing helmet use for motorcyclists and implementing car seatbelts, child passenger seat restrictions, speed limits, and drinking driving laws^31^. These initiatives have resulted in a noteworthy reduction in the mortality rate of TBI in China from 17.06 per 100 000 individuals to 12.99 per 100 000 individuals^32^. In LMICs, different types of helmets provide different levels of protection, and nonstandard helmets provide significant protection against TBI^35^. The implementation of helmet laws has proven effective in reducing motorcycle-related trauma^36^, resulting in a 41% reduction in TBI and an overall 29% decrease in motorcyclist fatalities^37^. In addition, wearing seatbelts while driving motor vehicles is one of the most effective ways to reduce the severity of injuries and incidence of fatalities. Patients wearing seatbelts generally have better prognosis^38^. The proportion of TBI caused by traffic accidents is high in LMICs, particularly in Africa and Southeast Asia (56%), and low in HICs, with the lowest proportion occurring in North America (25%)^39^. As the incidence of TBI-related to traffic accidents is expected to decrease further in China owing to policy changes and law enforcement, the incidence of TBI due to falls may become a predominant cause of the changing demographic patterns associated with population aging. This shifting epidemiological pattern is similar to that observed in HICs^40^. A study from the United States found that the number and rate of fall-related TBI in the elderly increases significantly between 2007 and 2013^41^. A research from New Zealand also indicated that falls are the leading cause of brain damage in people aged 65 or older^42^. Between 2008 and 2017, the age-adjusted death rate from falls increased by 17%, and the highest death rate from falls-related brain injuries was among the elderly^43^. Therefore, falls have gradually become the main cause of TBI in the elderly and TBI in the elderly becomes a key and hot research area^1,44^. In the United States, accidental falls are the most common cause of nonfatal craniocerebral injury-related hospitalization^45^. Additionally, the Global Burden of Diseases, Injuries, and Risk Factors (GBD) reported that falls are the most common cause of TBI globally with 52% in 1990, and 54% in 2019^3^. International efforts are underway to address TBI resulting from falls among older adults. Initiatives such as the STEADI (Stopping Elderly Accidents, Deaths & Injuries) (www.cdc.gov/STEADI) aim to reduce the risk of falls in this population^46^.

Although the current GBD studies suggest that the incidence of TBI in LMICs is higher than in HICs^1^, which is confirmed in the Global Neurotrauma Outcomes Study^47^. Rigorous national epidemiological studies are particularly scarce for TBI in LMICs and the assessing methodology is diverse^32^. This situation leads to the difficulties of comparison among the causes, incidences, and outcomes of TBI cross countries based on development level with the limited data.

Comprehensive epidemiological studies should be conducted worldwide to effectively prevent and control TBI. Response measures and targeted prevention strategies can be enhanced by understanding the specific weaknesses and challenges of the TBI prevention systems in different countries. Collaborative international efforts may play a crucial role in sharing the best practices, knowledge, and resources to reduce the global burden of TBI. Through cooperation, significant progress can be made to reduce the risk of TBI and improve the overall well-being of individuals and communities worldwide.

### Different monitoring methods and deep learning are progressing together

ICP monitoring significantly increased between 1990 and 2000^48^. In our study, ‘Intracranial pressure monitoring’ and ‘Deep learning’ topics are included in topic modeling. The ICP monitoring plays a crucial role in preventing the deterioration of TBI by providing information on ICP for the symptomatic treatment of TBI and by creating optimal conditions for brain functional recovery^49^.

However, the effectiveness of ICP monitoring in TBI remains controversial. Studies have shown that sTBI with ICP monitoring is associated with higher survival rate^50^. However, other studies concluded that ICP monitoring does not offer significant advantages over imaging-guided monitoring^51^. Recent studies have explored the integration of ICP monitoring with brain tissue oxygenation monitoring to reduce mortality^52^. Several trials have been conducted to determine the benefits of oxygen-targeted management^15^. Further research into the pathophysiological mechanisms of TBI is needed, quantifying temporal relations and dependencies of multimodal approaches, and enabling more effective and rapid monitoring strategies^53^. Assessing outcomes through multimodal monitoring is crucial for advancing future surgical and nonsurgical strategies for TBI treatment^54^.

The incorporation of deep learning methods into multimodal detection can reduce the burden on healthcare workers and enhance the accuracy of TBI monitoring. Deep learning has emerged as a powerful tool for distinguishing patients with mTBI from healthy individuals, particularly for identifying differential features using neuroimaging data^55^. Recent studies have demonstrated that deep learning methods can effectively identify patients with mTBI using various imaging modalities, such as CT, MRI, and diffusion tensor imaging^55–58^. Furthermore, deep learning can be used to predict the prognosis of TBI. For example, in pediatric TBI, deep learning assists in predicting the need for CT examinations and offers insight into prognosis^59,60^. In recent years, the prediction of trauma prognosis through the development of deep learning feature libraries has gained significant attention^61–63^.

The integration of deep learning methods and multimodal monitoring holds great promise for enhancing TBI diagnosis, prognosis prediction, and treatment planning. The application of these techniques may contribute to improved clinical decision-making and patient outcomes in the field of TBI.

### The use of biomarkers for mTBI can be multidirectional

Although ‘Biomarkers’ shows a decreasing trend over the past 5 years in our study, research in this field is still important for the diagnosis of mTBI, which not only poses a great sociality burden, but also affects functional recovery and their quality of life^64^. For mTBI with a negative CT examination, biomarkers can be used for rapid diagnosis and have some predictive ability for prognosis. Therefore, to understanding biomarkers is very important in this field. The initial biomarker studied for TBI was S100B^65^; however, its lack of specificity^66^ and limited predictive value for mTBI prognosis hindered its clinical use^67^. Emerging research on ubiquitin C-terminal hydrolase-L1 (UCH-L1) and glial progenitor fibrillary acidic protein (GFAP) is promising in this area^67–69^. Studies have indicated that UCH-L1 and GFAP levels are highly correlated with severe disability and mortality in patients with sTBI^70,71^, which is consistent with the results of another study on TRACK-TBI^72^. A low plasma concentration of UCH-L1 is associated with a favorable prognosis^73^, although its clinical value may be limited to sTBI^74^. In contrast, GFAP can predict time to return to work in patients with mTBI^66,67^. Similarly, a study on CENTER-TBI showed that elevated serum GFAP levels were strongly predictive of imaging abnormalities after TBI^75^, and another study on TRACK-TBI provided the same conclusion^76^. In recent years, research has focused on tau protein^77,78^ and neurofilament light chains as biomarkers of TBI^79,80^, which can predict the severity of symptoms after concussion and diagnose subacute and chronic TBI, respectively. These biomarkers hold great promise for the early diagnosis and treatment of mTBI and chronic traumatic encephalopathy (CTE).

### Contradictions in the treatment of TBI need to be resolved

Secondary brain injuries, including cerebral ischemia, hypoxia, inflammatory reactions, metabolic dysfunction, and cerebral edema, can occur after TBI^81^. ‘Therapeutic hypothermia’ (TH) is included in our topic model and has been investigated as a neuroprotective approach to mitigate neuronal damage and reduce neuronal loss during TBI^82^. Although there is a significant body of research on TH, its effect on patients remains unclear^83^. Randomized clinical trials conducted since 1990s have yielded conflicting findings regarding the therapeutic effects of TH on TBI. Some studies suggest that TH accelerates neurological recovery and improves outcomes in patients with TBI^84,85^. However, contradictory evidence still exists, as some studies have indicated that prolonged TH reduces the risk of death^86^, whereas early TH is not beneficial for sTBI^87,88^. Criticisms of previous findings highlight that the potential reason for the contradictory results is the silent reduction in cerebral oxygenation in patients with TBI^82^. A prospective, multicenter, randomized controlled clinical trial conducted in China revealed that prolonged mild hypothermia improved neurological outcomes and mitigated adverse outcomes in patients with sTBI and an initially high ICP^89^. Therefore, further research is necessary to elucidate the potential and appropriate applications of TH in TBI management.

Decompressive craniectomy (DC) is another intervention used to alleviate the elevated ICP in patients with TBI. It effectively reduces ICP and increases cerebral perfusion pressure^31,90^. Early and extensive DC has garnered interest among neurosurgeons^91,92^, and studies have suggested that it may be indicated for TBI treatment^93^. Although DC can lower ICP and reduce ICU length of stay, it can also lead to significant side effects such as the development of a vegetative state^94,95^. Consequently, DC is often considered a life-saving, last resort procedure for patients with sTBI and bilateral diffuse cerebral edema^96,97^. Determining the optimal timing and indications for DC in patients with medically refractory elevated ICP requires further investigation.

Management of elevated ICP plays a crucial role in TBI prognosis, and reducing the brain water content has long been recognized as an effective means of controlling ICP. Hypertonic fluids such as mannitol can restore cerebral perfusion, reduce cerebral edema, and modulate the inflammatory response to minimize neuronal damage, offering potential benefits for the resuscitation of patients with TBI^98^. Mannitol, a commonly used hypertonic solution for high ICP treatment in patients with TBI, effectively reduces brain tissue water content and lowers ICP^99,100^. Hypertonic saline (HS) has emerged as an alternative to mannitol, and some studies have suggested its superiority in reducing elevated ICP^101–103^. However, other studies have concluded that HS and mannitol are equally effective^104,105^. Conflicting findings prompted two meta-analyses published in 2011, which indicated that HS might be more effective than mannitol in treating elevated ICP, highlighting the potential adverse effects of mannitol, such as osmotic diuresis-induced hypotension and impaired renal function^106,107^. In 2021, a multicenter randomized clinical trial concluded that the continuous infusion of HS did not improve the neurological functional status of patients with TBI^108^. Therefore, further investigation is necessary to determine the most effective treatment strategy for hypertonic fluids.

### Pharmaceutical development can improve the prognosis of TBI

Drug research plays a crucial role in the treatment of TBI, one of the topics that result from our topic-modeling analysis. Intracranial hemorrhage is a common complication in patients with TBI and can occur or worsen during treatment^109^. Tranexamic acid (TXA), an antifibrinolytic agent, has been extensively studied for its potential to reduce cerebral hemorrhage after TBI^110^. In recent years, international research on the use of TXA for the treatment of cerebral hemorrhage after TBI has gained significant attention. Roberts *et al*.^111^ investigated the role of TXA in TBI, and their findings indicated that TXA reduced the risk of death in patients with mTBI and was particularly effective in patients with less severe injuries^112,113^.

Progesterone reduces brain edema after TBI^114^. Phase II randomized controlled trials conducted in 2007 and 2008 demonstrated that progesterone improved patient prognosis^115,116^; however, a subsequent prospective phase III randomized clinical trial concluded that progesterone did not provide benefits in sTBI^117^.

### Chronic traumatic encephalopathy still requires autopsy for diagnosis

CTE appears in the topic modeling of the top 30 citation journal data, indicating that this topic receives attention from researchers. CTE is a special type of TBI^118–120^ that requires autopsy for diagnosis. It was initially described in the 1960s as a syndrome observed in boxers, with movement disorders and mental confusion, commonly known as ‘punch-drunk’^121^. Modern understanding of CTE began in 2005 with an autopsy report by Omalu *et al*., who identified CTE neuropathology in a patient with a chronic mood disorder^122,123^.

CTE is a progressive neurodegenerative disease characterized by brain and medial temporal lobe atrophy, ventriculomegaly, an enlarged hyaline septum, and extensive tau-immunoreactive lesions throughout the neocortex, medial temporal lobe, mesencephalon, brainstem, and spinal cord. The defining lesion of CTE is the abnormal accumulation of hyperphosphorylated tau (p-tau) in neurons, with astrocytes distributed irregularly around small vessels deep in the cortical sulcus. Clinically, CTE is associated with memory impairments, behavioral and personality changes, Parkinson’s syndrome, and speech and gait abnormalities. CTE may present with two distinct clinical patterns: one characterized by initial behavioral or mood changes and the other by initial cognitive impairment^124^. However, caution is needed when diagnosing CTE in patients with a history of head trauma and neurocognitive decline, as other neurodegenerative disorders may also be present^125^. In 2015, a study demonstrated an association between amyloid β peptide (Aβ) deposition and more severe tau pathology in CTE, suggesting that Aβ may accelerate tauopathy^126^. Currently, CTE can only be definitively diagnosed postmortem, and efforts to diagnose it during a person’s lifetime remain challenging. Furthermore, effective prevention, treatment, and cure of CTE are yet to be developed^127^.

### Limitation

Our study had several limitations. First, our analysis was based solely on the WOSCC database, which might have resulted in exclusion of relevant articles from other databases. In addition, only articles published in English were included in our study; therefore, valuable information published in other languages might have been overlooked. Second, it was impossible to verify each document since the vast quantity of literature was included; therefore, the risk of errors and omissions in the raw data from a computational standpoint was unavoidable. However, our subsequent manual analysis can partially rectify this issue. Third, the full name and abbreviation of the same author or institution might vary in the different periods or journals. An author might change research institutions, which also caused confusion. Entity alignment (institution and author) is a current problem in all bibliometric analyses. We checked the results later to make up for it as much as possible. Fourth, some high-quality articles published recently might not have been fully cited due to citation delay. Finally, the topic naming in topic modeling analysis based on the experiences of professors might result in a minor discrepancy in the interpretation of topics.

## Conclusions

Over the past 20 years, TBI has attracted significant research attention. By using deep learning algorithms, specifically LDA, the current popular topics in TBI research were summarized. Although efforts have been made by many countries to prevent TBI, the field still faces challenges owing to its heterogeneity and variations in diagnosis and treatment. The incidence and mortality rates of TBI have remained largely unchanged over the past two decades. The risk causes of TBI have gradually changed from traffic accidents to falls, especially in HICs, which might be related to the aging population. Our study serves as a valuable resource for identifying research gaps and fostering multidisciplinary collaborations. Epidemiological studies have increased in number and become a direction for future breakthroughs. Overall, our study provides valuable insights into the current state of TBI research and highlights the need for continued efforts and collaboration to effectively address this significant public health issue.

## Ethical approval

Not applicable.

## Consent

Not applicable.

## Sources of funding

This study was funded by Liaoning Provincial Natural Science Foundation (2020-MS-155), China Medical University novel coronavirus pneumonia prevention and control research project (2020-12-11), Shenyang Planning Foundation for Science and Technology (21-173-9-38), the first batch of medical education scientific research project of China Medical University for the 14th Five-Year Plan (YDJK2021011), National Science Foundation of China (72074104), Immersive Smart Devices for Healthcare System R&D and Industrial Application Innovation Platform (2022) of Immersion Technology and Evaluation Shandong Engineering Research Center (2022), Research Project on Undergraduate Teaching Reform of Liaoning General Higher Education sponsored by Educational Department of Liaoning Province (2022-10159-479), Research Project on Postgraduate Teaching Reform sponsored by Educational Department of Liaoning Province (2022-10159-311), China Stroke Association Whole Course Management of Cerebrovascular Disease Sailing Fund (202001), 2023 Undergraduate Teaching Reform Research Project of China Medical University - 2022 Provincial First-class Curriculum Construction Specialization, and The Natural Science Foundation of Liaoning Province of China (2021-MS-179). The researchers are grateful for the support of several organizations.

## Author contribution

Dr Bao had full access to all the data in the study and took responsibility for the integrity of the data and the accuracy of the data analysis. Z.Y., Z.L., X.S., and Y.B.: concept and design and acquisition, analysis, or interpretation of data; Z.Y. and Y.B.: critical revision of the manuscript for important intellectual content; S.Z. and Q.X.: statistical analysis; K.L. and W.S.: supervision.

## Conflicts of interest disclosure

The authors declare that they have no competing interests.

## Research registration unique identifying number (UIN)

1. Name of the registry: not applicable.
2. Unique identifying number or registration ID: not applicable.
3. Hyperlink to your specific registration (must be publicly accessible and will be checked): not applicable.

## Guarantor

Yijun Bao.

## Data availability statement

Not applicable.

## Provenance and peer review

Not commissioned, externally peer-reviewed.

## Supplementary Material
