# Supplementary material for: The recent two decades of traumatic brain injury: a bibliometric analysis and systematic review
Source: Int J Surg. 2024 Apr 11;110(6):3745–59. doi: 10.1097/JS9.0000000000001367 (PMC11175772; doi:10.1097/JS9.0000000000001367)
Supplement: Supplementary file 1 [file js9-110-3745-s001.docx]

**Supplemental Digital Content**


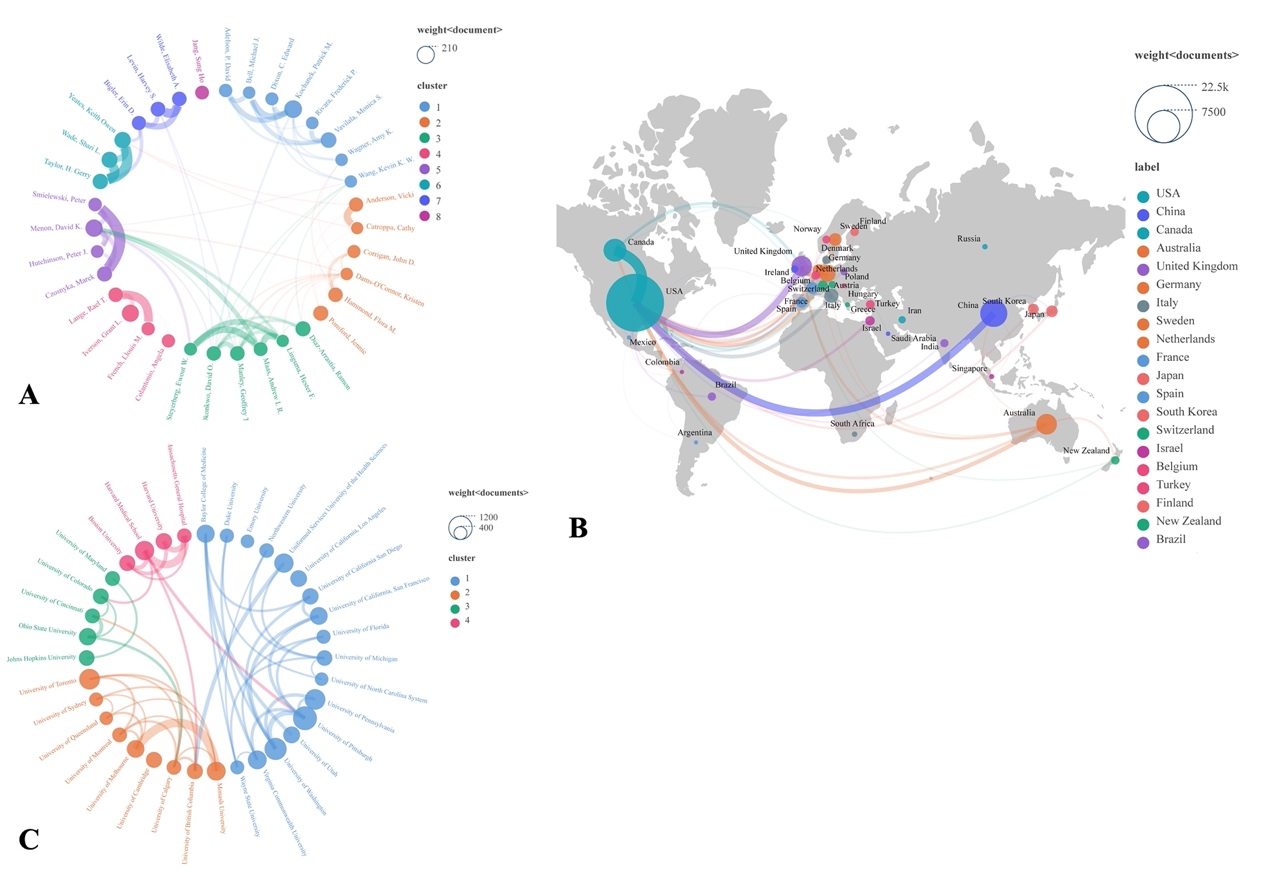


**SDC Figure 1** Coauthorship and cooperation analysis in the field of TBI. Node represents author, country, and institution respectively in A, B and C. **A:** Network visualization of coauthorship. **B:** Distribution of the countries/regions in terms of the number of publications on the world map. **C:** The cooperative network visualization between institutions. The different colors represent the categories classified by the collaborations.


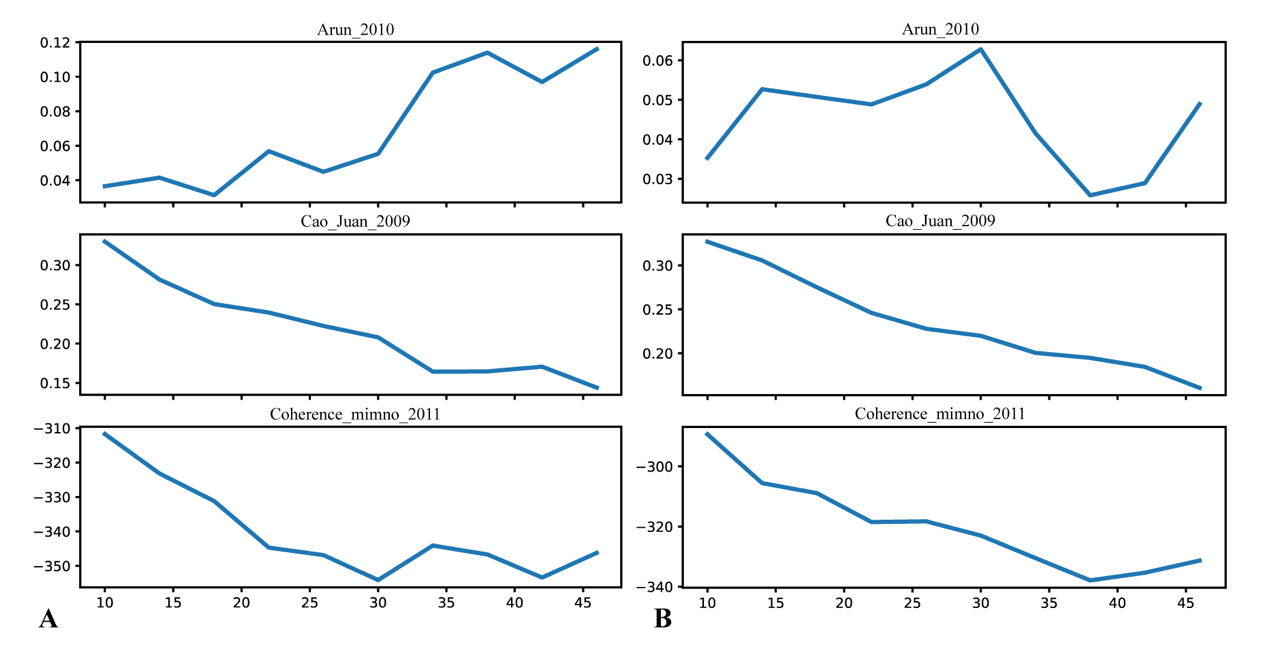


**SDC Figure 2** Metrics to select the number of topics. **A**: The total data; **B**: The data of the top 30 citation journals.

**SDC Table 1** Statistical analysis of the trends of topics by Mann-Kendall test for the total data.

| **Number** | **Topic** | **Z** | **P** |
| --- | --- | --- | --- |
| 1 | Rehabilitation | -4.3151 | 1.595e-05 |
| 2 | Alcohol | 3.9906 | 6.589e-05 |
| 3 | Animal experiments: Drug therapy | -4.5746 | 4.77e-06 |
| 4 | MRI | 0.68133 | 0.4957 |
| 5 | Animal model | -0.55155 | 0.5813 |
| 6 | Behavioral disorders | -2.1089 | 0.03496 |
| 7 | Neurodegeneration | 2.3035 | 0.02125 |
| 8 | Hospital management | 3.0822 | 0.002055 |
| 9 | Sports-related concussion | 4.8991 | 9.628e-07 |
| 10 | Prognosis predict model | 3.8609 | 0.000113 |
| 11 | Therapeutic hypothermia | -4.7693 | 1.849e-06 |
| 12 | Etiology | -2.3684 | 0.01786 |
| 13 | Clinical Trial | 2.4333 | 0.01496 |
| 14 | Biomarkers | -2.1738 | 0.02972 |
| 15 | Military-related TBI | 1.3302 | 0.1834 |
| 16 | Severity of trauma | 4.1853 | 2.848e-05 |
| 17 | Mild TBI | 1.5249 | 0.1273 |
| 18 | Deep learning | 3.6013 | 0.0003166 |
| 19 | Adolescent TBI | -3.9258 | 8.646e-05 |
| 20 | Neuroinflammation | 5.0938 | 3.51e-07 |
| 21 | Neuropsychology | -5.0938 | 3.51e-07 |
| 22 | Neurogenesis | -3.9258 | 8.646e-05 |
| 23 | Physical and mental disorders | -0.292 | 0.7703 |
| 24 | Social factors | -1.1355 | 0.2561 |
| 25 | Neuronal apoptosis | -3.7311 | 0.0001907 |
| 26 | Epilepsy | 0.81111 | 0.4173 |
| 27 | Helmet | 3.212 | 0.001318 |
| 28 | Cerebral hemorrhage | -0.81111 | 0.4173 |
| 29 | CT | 1.7195 | 0.08551 |
| 30 | Intracranial pressure monitoring | -3.6013 | 0.0003166 |

Note: TBI, traumatic brain injury; MRI, magnetic resonance imaging; CT, computed tomography.

**SDC Table 2** Statistical analysis of the trends of topics by Mann-Kendall test for the top 30 citation journal data.

| **Number** | **Topic** | **Z** | **P** |
| --- | --- | --- | --- |
| 1 | Deep learning | 3.1163 | 0.001831 |
| 2 | Axonal injury | -4.6395 | 3.492e-06 |
| 3 | Physical and mental disorders | 0.19477 | 0.8456 |
| 4 | Neuroinflammation | 2.3684 | 0.01786 |
| 5 | Neurogenesis | -4.1853 | 2.848e-05 |
| 6 | Animal experiments: Drug therapy | -4.7693 | 1.849e-06 |
| 7 | MRI | 1.7195 | 0.08551 |
| 8 | Animal model | 3.2769 | 0.00105 |
| 9 | Clinical Trial | 2.7578 | 0.00582 |
| 10 | Epilepsy | -0.22711 | 0.8203 |
| 11 | Rehabilitation | -1.1355 | 0.2561 |
| 12 | Cognitive impairment | -0.48666 | 0.6265 |
| 13 | Behavioral disorders | -0.35689 | 0.7212 |
| 14 | Sports-related concussion | 3.8609 | 0.000113 |
| 15 | Neuropsychology | -1.6547 | 0.09799 |
| 16 | Helmet | 0.94088 | 0.3468 |
| 17 | Chronic traumatic encephalopathy | 2.5969 | 0.009407 |
| 18 | Severity of trauma | 3.212 | 0.001318 |
| 19 | Mild TBI | 3.3418 | 0.0008325 |
| 20 | Microdialysis | -3.8609 | 0.000113 |
| 21 | Neuronal apoptosis | -1.8493 | 0.06441 |
| 22 | CT | 0.74622 | 0.4555 |
| 23 | Moderate to severe TBI | -1.5898 | 0.1119 |
| 24 | Intracranial pressure monitoring | -0.61644 | 0.5376 |
| 25 | Hospital management | 2.2387 | 0.02518 |
| 26 | Signaling pathway | -3.0822 | 0.002055 |
| 27 | Prognosis predict model | 1.46 | 0.1443 |
| 28 | Caring | 1.5898 | 0.1119 |
| 29 | Therapeutic hypothermia | -3.1471 | 0.001649 |
| 30 | Biomarkers | -0.876 | 0.381 |

Note: MRI, magnetic resonance imaging; TBI, traumatic brain injury; CT, computed tomography.
